# Chemical Constituents Involved in E-Cigarette, or Vaping Product Use-Associated Lung Injury (EVALI)

**DOI:** 10.3390/toxics8020025

**Published:** 2020-04-03

**Authors:** Thivanka Muthumalage, Michelle R. Friedman, Matthew D. McGraw, Gary Ginsberg, Alan E. Friedman, Irfan Rahman

**Affiliations:** 1 Department of Environmental Medicine, University of Rochester Medical Center, Box 850, 601 Elmwood Avenue, Rochester, NY 14642, USA; Thivanka_Muthumalage@URMC.Rochester.edu; 2 Department of Chemistry & Biochemistry, The College of Brockport, State University of New York, Brockport, NY 14420, USA; mfriedman@brockport.edu; 3 Division of Pediatric Pulmonology, University of Rochester Medical Center, School of Medicine & Dentistry, Rochester, NY 14642, USA; Matthew_Mcgraw@URMC.Rochester.edu; 4 New York State Department of Health, Center for Environmental Health, Albany, NY 12237, USA; Gary.Ginsberg@health.ny.gov; 5 Department of Materials Design and Innovation, School of Engineering and Applied Sciences, University at Buffalo, Buffalo, NY 14260, USA; alanfrie@buffalo.edu

**Keywords:** Vaping, EVALI, VAPI, e-cigarette, THC, cannabis, lipoid-pneumonia

## Abstract

The Centers for Disease Control declared e-cigarette, or vaping, product use-associated lung injury (EVALI) a national outbreak due to the high incidence of emergency department admissions and deaths. We have identified chemical constituents in e-cig counterfeit cartridges and compared these to medical-grade and CBD containing cartridges. Apart from vitamin E acetate (VEA) and tetrahydrocannabinol (THC), other potential toxicants were identified including solvent-derived hydrocarbons, silicon conjugated compounds, various terpenes, pesticides/plasticizers/polycaprolactones, and metals. This study provides additional insights into the chemicals associated with EVALI cartridges and thus may contribute to the underlying disease mechanism of acute lung injury.

## 1. Introduction

Electronic cigarette (e-cig) use or vaping has gained popularity in the past decade, especially among the youth, due to the availability of many enticing flavors and devices. In August 2019, CDC started reporting e-cigarette, or vaping, product use-associated lung injury (EVALI) cases. The majority of patients with EVALI report vaping tetrahydrocannabinol (THC)-containing counterfeit street e-cig products. By December 2019, there have been over 2,500 hospitalized EVALI patients, most of whom are men younger than 35 years of age [1]. Patients hospitalized with EVALI have manifested a variety of radiological imaging patterns consistent with lipoid pneumonia, eosinophilic pneumonia, and chemical damage to the lung tissue [2,3]. Patients diagnosed with this illness have reported symptoms, such as a cough, shortness of breath, chest pain, nausea, vomiting, diarrhea, fatigue, fever, or weight loss [4].

Counterfeit street cartridges are produced by using cutting agents, such as medium-chain triglyceride (MCT) oil, vitamin E (tocopherol) acetate (VEA), and seized drugs, such as butane hash oil [5]. Currently, vitamin E acetate (VEA) has been identified as a key agent involved in EVALI [6]. However, while VEA is present in cartridges and BALF from patients, VEA inhalation is not a proven cause of pneumonia or other types of lung damage and so other constituents, if present, should also be considered. Further, VEA may be protective against the toxic and inflammatory effects of lipopolysaccharide and cigarette smoke in the lung [7,8]. Thus, we hypothesized that there may be other constituents in these e-cig THC-containing cartridges that can act singularly, or in concert with VEA, inducing injuries to the lung.

To address this question, we have screened for chemical constituents in liquid and vapor phases from counterfeit street cartridges. We also identified and compared the chemical constituents in CBD-containing cartridges and medical-grade vaping cartridges, products not known to be associated with EVALI. Determining the underlying chemistry in each product type may help to understand the underlying mechanism of lung injury associated with vaping.

## 2. Methods

### 2.1. Ethics Statement on Biosafety Approval

All experiments performed in this study were approved and in accordance with the University of Rochester Institutional Biosafety Committee (Study approval #Rahman/102054/09-167/07-186; identification code: 07-186; date of approval: 01/05/2019).

### 2.2. Counterfeit Street/Patient, CBD-Containing, and Medical-Grade Cartridges

E-cig vaping counterfeit (bootleg/street), CBD-containing, and/or medical-grade cartridges (Columbia Care, TheraCeed, New York, NY, USA) were obtained from local vape shops and users/patients. The counterfeit street cartridge brands recovered from patients with EVALI (Kalininskiy et al 2019) included Dank Vape, ROVE, Super G Cookies, Runtz, Chronic Billy Kimber, Exotic (Paris OG), Jungle boys, Most Coast, Chronic Gushers, Chronic Gruntz, Jungle skittles, TKO, Clear Chronic, Exotic Carts, and Smart Carts. No manufacturers’ details were given on cartridges or packing materials. The tested variants of cannabis oil included *sativa, indica,* and *hybrid*. A medical-grade marijuana cartridge was used as a known reference material for comparison.

### 2.3. Screening for Chemical Constituents in E-Liquids by GCMS

Chemical constituents in counterfeit or patient-provided (*n* = 38), CBD-containing (*n* = 4), and medical-grade (positive) e-cig vaping cartridges were determined by gas chromatography (GC) and mass spectrometry (MS). Two sample types were analyzed: cartridge rinsates from e-cigarette cartridges and neat e-cigarette liquids. Rinsates were generated by flush cartridge interiors 5× with 100 μL chromatography grade methanol (Ricca chemical company, Cat #R4828000-1C, Arlington, TX, USA) combined into one sample and then diluted 100-fold in spectral grade methanol. Neat e-cigarette liquids were diluted 100-fold in spectral grade methanol and injected into the GCMS system, an Agilent 7890A gas chromatograph with 5975 MSD detector (Agilent Technologies, Santa Clara, CA, USA). The system used helium as the carrier gas, flowing 1.2 mL/min through an Agilent Technologies column (HP-5MS, 30 m × 0.250 mm, 0.25µm, 19091S-433). The oven program initiated at 45 °C for one minute, ramped to 285 °C over seven minutes at 10 °C/min, ramped to 300 °C for ten minutes at 10 °C/min, and finally ramped a third time to 325 °C over five minutes at 5 °C/min. The total run time was 53.5 minutes, and the injection volume was 1µL from a 10µL syringe. The samples were analyzed by electron impact ionization in positive ion mode with a mass range of 50–550 *m*/*z*, with the source temperature at 230 °C and the quadrupole at 150 °C. Data analysis was performed using Agilent ChemStation software (MSD ChemStation, E.02.02.1421, Agilent Technologies, Santa Clara, CA, USA), with ion scans searched against The NIST Mass Spectral Search Program database (Version 2.1.2.7, National Institute of Standards and Technology, Gaithersburg, MD, USA) for identification.

Chemicals commonly found in each category (counterfeit, CBD-containing, and medical-grade) of e-cig cartridges, but not found in other categories, were identified and classified into terpenes, silicon compounds, pesticide constituents, flavor additives, cannabinoids, plasticizers/polycaprolactones (PCP)/drugs/manufacturing agents, humectants/oil/plant components, vitamin and conjugates, and miscellaneous constituents (Table 1).

### 2.4. Quantifying Vapor Phase Chemical Constituents

Aerosols from e-cig vaping cartridges were sampled in 1L vacuum bottles, and each cartridge was sampled for ten minutes with ten puffs each. These samples were sent to ALS Environmental, CA, for analysis. Constituents remaining in the vapor phase after storage and shipping were analyzed by EPA method TO-15 which focuses upon a standard suite of terpenes and volatile organic compounds (VOCs). In addition, a mass spectral library search was used for tentatively identified compounds. The most abundant compounds were then classified into groups based on their use, i.e., terpenes, manufacturing/pesticides, automotive, solvents, PCP/household, and miscellaneous constituents (Table 2).

### 2.5. Elemental Analysis of Cartridge Liquids

To screen for elements of liquid aliquots from CBD-containing and counterfeit/patient-provided cartridges, ICP-MS analysis was performed. Total Quant KED analysis of elements used NeXION 2000 ICP-MS (PerkinElmer, Waltham, MA, USA) with external standards (20 ppb) solution in 2% nitric acid of 50 elements, adding helium gas at a rate of 4 mL/min. The solutions were pumped into a Meinhard nebulizer cooled to 2°C. This generated an aerosol that was aspirated into the plasma torch, where ionization occurs. The ion beam was then detected by the mass spectrometer. The most abundant elements in the liquids, particularly metals were identified.

## 3. Results

### 3.1. Presence of Unique Chemicals in Counterfeit Patient-Provided, CBD-Containing, and Medical-Grade E-Cig Vaping Cartridges

More than 500 chemicals were found in tested cartridges. The chemical constituents peculiarly present in e-cig counterfeit, patient-provided, CBD-containing, and medical-grade cartridges are listed in Table 1. The majority of the compounds in e-cig cartridges were terpenes and silicon compounds. Chemicals unique to e-cig counterfeit patient-provided specific cartridges include 2,2-dimethoxybutane, tetramethyl silicate, decane, methyl esters, ethyl esters, siloxanes, and acetates, including α-tocopherol/vitamin E acetate (VEA). Apart from the chemicals listed above, e-cig counterfeit patient cartridges consisted of many terpenes and their acetates. Other compounds present included benzophenone, glycerol tricaprylate and similar products, and THC and its derivatives.

In most e-cig vaping CBD-containing cartridges, cannabidiol was present as opposed to THC. Glycerin, siloxanes, and flavoring chemicals, such as ethyl vanillin and coumarin compounds, were found in CBD-containing e-cig cartridges.

In e-cig medical-grade cartridges, acetyl chloride, vitamin A retinol-conjugated compounds, such as retinol acetate, rather than VEA, were present.

### 3.2. Vapor-Phase Constituents in Counterfeit E-Cig Cartridges

By screening for the vapor-phase constituents in counterfeit patient cartridges, we identified and quantified over 50 chemicals with concentrations ranging from 140 µg/m^3^ to 360,000 µg/m^3^ (Table 2). Predominantly these vapor-phase constituents were manufacturing/pesticides/automotive/industrial active and inert agents and solvents.

### 3.3. Metal Constituents in E-Liquids

Elemental analysis of the e-liquids from THC-containing, counterfeit, patient-provided cartridges was performed. In counterfeit patient-cartridges, the most abundant elements included Si, Cu, Ni, and Pb. These metal concentrations were highly variable between cartridges, though the concentrations reached as high as 600 ppm.

## 4. Discussion

In this study, we investigated the chemical constituents commonly found in e-cig vaping patient-provided counterfeit cartridges, CBD-cartridges, and medical cartridges. The presence of harmful compounds, such as MCT oil, VEA, and other lipids in THC-containing cartridges has been identified by the FDA/CDC [9]. At present, VEA has been linked most consistently to EVALI cases based on its presence in (BALF) from such cases [6]. Thus far, the EVALI epidemic has only been seen in North American populations, with very few cases reported in Canada [10]. Interestingly, a recent study on e-cigarette ingredient analysis reported that there is no VEA in vaping products in the UK [11].

We determined numerous constituents in counterfeit-patient, CBD, and medical-grade e-cig vaping cartridges. We found several solvent chemicals, such as n-butane and a derivative, 2,2 dimethoxybutane, in both liquid and vapor phases of counterfeit e-cig cartridges. This is likely a result of “dabbing” with butane hash oil. Dabbing is an extraction procedure commonly practiced in making illicit street cartridges as shown to improve total THC-recovery and lung availability by greater than 70%, which is unachievable by smoking cannabis alone [12]. Inhaling butane hash oil derivatives have already been seen as a probable cause for atypical/eosinophilic pneumonia in case reports [13,14].

The presence of hydrocarbons, such as decane and undecane, can also cause central nervous system damage, respiratory irritation, and even induce chemical pneumonitis [15]. Similar to the effects of aspirating kerosene, these hydrocarbon solvents may induce pulmonary edema, cause lesions, destruct alveolar and capillary membranes, and alter surfactant production and function [16]. Indeed, the total volatile organic compounds (VOC) were emitted higher in counterfeit patient cartridges (20.03 ± 0.59 ppm) versus those emitted by MCT (10.33 ± 0.88 ppm), Mineral oil (7.33 ± 0.88 ppm), and VEA (9.67 ± 0.33ppm), means ± SE as monitored by QTrack VOC monitor. Further, their particle concentrations (mg/m^3^) in aerosols were also higher measured as particulate matter (diameter 1.0, 2.5, and 10 μm) in counterfeit patient cartridges vs MCT, mineral oil, and VEA, by Dustrack II 8530 instrument. Interactions between these hydrocarbons, particulates and other organic oils, such as mineral oil, MCT oil, and coconut oil, may promote or delay the absorption and metabolism of these compounds [17]. However, given the nature of the disease manifestation of the reported cases of EVALI with neutrophil and eosinophil infiltration in the BALF, and their successful treatment with steroids, most of the cases may have been chemical-induced pneumonitis [2,18].

In our chemical analysis, many conjugates and derivatives of silicon, such as silicates, silanes, and siloxanes, were identified only in e-cig counterfeit cartridges. Moreover, we also found large amounts of silica during elemental screening. These compounds, such as tetramethyl silicates, can form secondary products, such as silicon dioxide (SiO_2_) and methanol, which are highly toxic. Inhalation of silicon compounds is known to cause acute lung injury with pulmonary edema and lesions [19].

It is believed that various hydrocarbons and reactive aldehydes are formed when e-liquids are heated to high temperatures around 500 °F/260 °C. All these cartridges, including CBD containing cartridges, are used at the common voltages (e.g. 3.5V to 5.5 V) using a specific device [20]. These conditions were used to generate aerosols for the detection of chemical constituents. Among the vapor-phase constituents, we found numerous known respiratory and cardiac toxicants specifically in e-cig counterfeit cartridges that are listed as harmful or potentially harmful constituents in tobacco products and tobacco smoke (HPHC) by the FDA. These compounds found in e-cig counterfeit cartridges include isoprene, acetaldehyde, ethylbenzene, toluene, acrolein, 1,3-butadiene, and benzene [21]. We did not find VEA in the vapor phase, but this may be due to the time lag between collection and analytical analysis given that at room temperature for several days any aerosolized VEA would have condensed onto the sides of the one liter cannister. Despite this limitation, our detection of a wide array of compounds in vaping aerosols from EVALI-associated products points to chemical constituents of potential concern in addition to VEA.

Even though we detected VEA in many EVALI-associated e-liquids, we did not see VEA in all such products. However, that does not mean that the other cartridges patients may have been using prior to admission were not VEA-containing. We tested what was provided and this was not necessarily a complete account of what was used prior to lung injury. Interestingly, medical-grade cartridges (formulation in coconut oil) contained retinol (vitamin A) acetate rather than VEA. Though there were still harmful chemicals, such as acetyl chloride, benzene, aflatoxin b1 identified in medical-grade cartridges, the number of contaminants detected in these cartridges were fewer compared to e-cig counterfeit patient-provided cartridges.

In counterfeit patient cartridges, we also found a common constituent in nasal sprays, oxymetazoline (decongestant), an α_1_ adrenergic receptor agonist. Common pesticide/insecticide ingredients, such as naphthalene and hexadecanoic, methyl ester, were also detected in liquid and vapor phases of these e-cig counterfeit /patient cartridges.

In the vapor-phase of e-cig counterfeit street cartridges, we detected many respiratory depressants and paralytic agents, such as 4-methyl-2 pentene, acetaldehyde, ethylbenzene, xylenes, and 1,3 pentadiene. Inhalation of these agents is potentially the cause of symptoms, such as dyspnea and chest tightness (common symptoms among EVALI patients). Inhalation of compounds, such as acetone and 1,2, dichloroethane, can cause drowsiness, which is another common symptom among EVALI patients. Among the vapor phase chemicals, inhalation of alkanes, such as n-butane, may cause acute lung toxicity. Moreover, exposure to toxicants such as methyl vinyl ketone and allyl chloride is highly toxic and causes emphysema, edema, and even vision impairment. The ability of any of these constituents to cause or contribute to lung injury will depend upon exposure conditions (concentration and duration).

Chemical constituents in counterfeit cartridges, e.g., 1,3-butadiene, can self-polymerize and form peroxides upon exposure to oxygen. These chemicals may react with other compounds and further be catalyzed into forming secondary products [22]. Inhaled xenobiotics undergo metabolism via phase I (CYP450/monooxygenase) and phase II (transferases) forming metabolites and detoxification/elimination products. During these processes, toxic metabolites may be formed. For example 1,3-butadiene has possible intermediates that are DNA reactive metabolites, such as butadiene monoepoxide and butadiene diepoxide [23]. Aside from the detected e-liquid chemical constituents, the lipid derivatives from the ‘endogenous’ source, such as the epithelial lining fluid (ELF) and/or lung surfactants by interacting with exogenous hydrocarbons with phospholipids and surfactants of the ELF, may also occur. This may be associated with the inflammatory responses using these counterfeit cartridges seen in the lungs of patients with EVALI [20]. It remains to be seen whether the identified chemicals have any role for pathological changes in the lung, such as centrilobular ground-glass nodules and ground-glass opacities with subpleural sparing, as seen in patients with EVALI.

One of the limitations of this study includes the qualitative nature of the found constituents/toxicants in cartridge liquids, as the GC-MS method established for this study was for preliminary screening to determine the presence of all constituents. Further, the samples obtained for testing came from a particular region of NYS and it is possible that other parts of the US may have different counterfeit cartridges involved in EVALI. A centralized library of EVALI-associated vape products from around the country would be very helpful in broadening the current analytical results, including chemicals identified in those cartridges for toxicological studies. Contributing susceptibility factors for EVALI pathogenesis may include genetic, environmental, lifestyle factors and concomitant diseases as not all THC-cartridge users were hospitalized for EVALI [24]. The use of other vaping products, such as flavored e-liquids and pods with nicotine salts, can be contributing and exacerbating factors in the pathogenesis of EVALI [3,25].

The legal restrictions on cannabis products limit research avenues to study the effects of these e-cig vaping products using surrogate models. Thus, there is a paucity of risk assessment and toxicological data on THC/cannabis containing products. Researchers and federal health organizations, such as the Centers for Disease Control (CDC) need to develop standardized protocols for e-cigarette research studies and clinical trials, which may involve modified human subject notification and approval protocols to participate in this type of research.

In conclusion, our chemical analysis in e-cig counterfeit street/patient THC-containing cartridges showed numerous respiratory toxicants in liquid and vapor phases that were not present in the medical or CDB vape products. Further, a number of symptoms observed in EVALI patients are similar to what can be anticipated from inhaling the detected compounds. The potential toxicants include solvent-based volatile (e.g., butane) and semi-volatile (e.g., decane, naphthalene) hydrocarbons, silicon conjugated compounds, various terpenes, pesticides/plasticizers/polycaprolactones and metals. Inhaling these chemicals at sufficient concentration is known to cause symptoms, such as a cough, shortness of breath, chest pain, nausea, vomiting, diarrhea fatigue, fever, or weight loss, consistent with that seen in patients with EVALI [4]. Our data suggest that exposure to a combination of hydrocarbons and oils, along with other toxic chemical compounds and metals, may contribute to the lipoid pneumonia potential effects of VEA and contributing causes of EVALI as opposed to a singular causative agent. We are further investigating this using various in vitro and in vivo models.

## Figures and Tables

**Table 1:** Commonly present constituents in counterfeit, CBD, and medical-grade cartridges.

Counterfeit Patient Cartridges	CBD-Cartridges	Medical-Grade Cartridges
**Terpenes**		
6,6-dimethyl-2-methylene-,[1S]-bicyclo[3,1,1]heptane	1,6-Octadien-3-ol, 3,7-dimethyl-	Azulene, 1,2,3,5,6,7,8,8a-octahydro-1,4-dimethyl-7-(1-methylethenyl)-, [1S-(1alpha,7alpha,8abeta)]-
Beta-pinene		1H-cyclopenta[1,3]cyclopropa[1,2]benzene, octahydro-7-methyl-3-methylene-4-(1-methylethyl)-, [3aS-(3aalpha,3bbeta,4beta,7alpha,7alphaS*)]-
Bicyclo[3.1.1]heptane, 6,6-dimethyl-2-methylene-, (1S)-		Decanoic acid, 1,1a,1b,4,4a,5,7a,7b,8,9-decahydro-4a,7b-dihydroxy-3-(hydroxymethyl)-1,1,6,8-tetramethyl-5-oxo-9aH-cycloprop
β-Myrcene		
D-Limonene		
Cyclohexene, 1-methyl-4-(1-methylethylidene)-		
1,6-Octadien-3-ol, 3,7-dimethyl-		
3-Cyclohexene-1-methanol, α,α4-trimethyl-		
Caryophyllene		
α-Caryophyllene		
Caryophyllene oxide		
Phytol		
Squalene		
**Silicon compounds**		
Cyclohexasiloxane, dodecamethyl-	1-butyl[dimethyl]silyloxypropane	1,3-bis(trimethylsiloxy)benzene
Cycloheptasiloxane, tetradecamethyl-	Dodecamethyl-cyclohexasiloxane	
Hexadecamethyl-cyclooctasiloxane	Cycloheptasiloxane, tetradecamethyl-	
Cyclononasiloxane, octadecamethyl-	Hexadecamethyl-cyclooctasiloxane	
Cyclodecasiloxane, eicosamethyl-	Octadecamethyl-cyclononasiloxane	
Octasiloxane, 1,1,3,3,5,5,7,7,9,9,11,11,13,13,15,15-hexadecamethyl-		
Tetramethyl silicate		
Benzyloxy(butyl)dimethylsilane		
Silane, (1,1-dimethylethyl)dimethyl(phenylmethoxy)-		
Cyclotrisiloxane, hexamethyl-		
Cyclooctasiloxane, hexadecamethyl-		
1,1,3,3,5,5,7,7,9,9,11,11,13,13,15,15-hexadecamethyl-octasiloxane		
**Pesticide constituents**		
Decanoic acid, methyl ester		
Hexadecanoic acid, methyl ester		
**Flavor additives**		
Calarene epoxide	3-ethoxy-1,2-propanediol	Nonanoic acid, methyl ester
Furo[2,3-H]coumarine, 2-(1-hydroxyethyl)-1,6-dimethyl-	Ethyl vanillin	10-undecynoic acid, methyl ester
Octanoic acid, methyl ester	1,6,10-dodecatrien-3-ol, 3,7,11-trimethyl-, [S-(Z)]-	Ethyl cyclohexapropionate
**Cannabinoids**		
δ9-Tetrahydrocannabivarin	6H-Dibenzo[b,d]pyran-9-methanol, 6a,7,8,10a-tetrahydro-1-hydroxy-6,6-dimethyl-3-pentyl-	
1,3-Benzenediol, 2-(3,7-dimethyl-2,6-octadienyl)-5-pentyl-		
1H-4-Oxabenzo(f)cyclobut(cd)inden-8-ol, 1a-α,2,3,3a,8b-α,8c-α-hexahydro-1,1,3a-trimethyl-6-pentyl-		
**Plasticizer/Polycaprolactone/drugs/manufacturing**		
Benzophenone		1,6,10-dodecatriene, 7,11-dimethyl-3-methylene-, (Z)-
Hexadecanoic acid, methyl ester		Pyrrolidine, 1-acetyl
Benzoic acid, 4-ethoxy-, ethyl ester		Cyclopenta[c]furo[3',2'4,5]furo[2,3-h][1]benzopyran-1,11-dione,2,3,6a,9a-tetrahydro-4-methoxy-, (6aR-cis)
Oxymetazoline		
Glycerol tricaprylate		
Pregn-9(11)-en-20-ol-3-on-19-oic acid lactone		
Pregna-5,15-dien-20-one, 3-hydroxy-16-methyl-,(3beta)		
**Humectacts/Oil/ Plant components**		
Ribitol, 1,3:2,4-di-O-benzylidene-	R-(-)-1,2-propanediol	5-hepten-2-one, 6-methyl
Decanoic acid, 1,2,3-propanetriyl ester	Diglycerol	Benzene, 1,3-bis(1,1-dimethylethyl)-
12-oleanen-3-yl acetate, (3alpha)-	Glycerin	Octanoic acid, ethyl ester
**Vitamins and conjugates**		
2H-1-Benzopyran-6-ol, 3,4-dihydro-2,5,7,8-tetramethyl-2-(4,8,12-trimethyltridecyl)-, acetate, [2R-[2R*(4R*,8R*)]]-		Retinol, acetate
Vitamin E		
**Miscellaneous**		
2,2-Dimethoxybutane		Acetyl chloride
Undecane		

**Table 2:** Vapor phase constituents in counterfeit cartridges.

Terpenes	Manufacturing/Pesticides/Automotive	Solvents	PCP-Polycaprolactone/ Household	Miscellaneous
Isoprene* [8400–360000]	4-Methyl-2-pentene [110000]	Ethanol [1500–4200]	1,3-Cyclohexadiene [33000]	1,3-Pentadiene [94000]
Camphene [5300]	1-Pentene [88000]	Acetone [4500–39000]	3,3-Dimethyl-1-butene [22000]	1,4-Pentadiene [57000]
β-Pinene [1400]	Sulfur dioxide [380]	Ethylbenzene* [600]	α –Fenchene [980]	C5H6 Compound [19000]
β-Myrcene [1100]	C5H10 Compounds [630–99000]	Toluene-d8 [510–516]	Acetaldehyde* [610–16000]	C6H12 Compound [19000–42000]
p-Isopropyltoluene [810]	n-Butanal [440]	2-Propanol [1300]	Methacrolein [550–24000]	2,4-Hexadiene [15000]
(+)-4-Carene [580]	C6H10 compounds [590]	1,2-Dichloroethane-d4 [391–395]		n-Pentane [1300]
γ –Terpinene [430]	n-butane [960]	Toluene* [210–20000]		Methyl vinyl ketone [580]
D-Limonene [1100]	Benzene* [97–16000]	Tetrahydrofuran [340]		Bromofluorobenzene [556–561]
α-Pinene [5300]	1,3,5-Trimethylbenzene [610]			Acrolein* [350–150000]
	o-Xylene [390]			2-Butanone (MEK) [94–3000]
	m,p-Xylenes [10000]			1,4-Dioxane
	1,3-Butadiene* [98–85000]			trans-1,2-Dichloroethene
	n-Hexane [44–610]			n-Heptane [140–2800]
	2-Methylpropene [2600–290000]			Propene [1500–300000]
	1-Hexene [120000]			α-Phellandrene[370]
	Propyne [23000]			

Concentration listed as a range within brackets in µg/m^3^. * Constituents classified as harmful or potentially harmful constituents in tobacco products and tobacco smoke (HPHC) by the FDA.
